# Spontaneous haemorrhagic stroke complicating severe pre-eclampsia in pregnancy: a case report in a resource-limited setting in Cameroon

**DOI:** 10.1186/s12884-018-2157-7

**Published:** 2018-12-27

**Authors:** Paul Nkemtendong Tolefac, Nkemnji Standley Awungafac, Jacqueline Ze Minkande

**Affiliations:** 1 Faculty of Medicine and Biomedical Sciences, University of Yaoundé 1, Yaoundé, Cameroon; 2 Mbalmayo District Hospita, Mbalmayo, Cameroon

**Keywords:** Haemorrhagic stroke, Pre-eclampsia, Pregnancy, Severe preeclampsia, Case report

## Abstract

**Background:**

Spontaneous intracerebral haemorrhage is a rare complication of preeclampsia during pregnancy associated with a high morbidity and mortality. Compared with the non-pregnant women stroke rates are relatively rare during pregnancy.

**Case presentation:**

We report the case of a 32-year-old female Cameroonian gravida 4 para 3 who presented at 34 weeks of gestation with sudden onset of right sided hemiplegia associated with headache, blurred vision and a blood pressure of 182/126. Cerebral CT scan confirmed a left parietal spontaneous haemorrhage. Emergency caesarean delivery was done and the recovery uneventful.

**Conclusion:**

This case highlights the importance of good neurological examination in pregnant women presenting with neurological symptoms as well as the place of multidisciplinary management in severe life threatening conditions.

## Background

Stroke is a clinical syndrome of rapidly developing clinical signs of focal (or global) disturbance of cerebral function, lasting more than 24 h or leading to death, with no apparent cause other than vascular origin [1], whereas intracerebral haemorrhage which is a type of stroke refers to rapidly developing clinical signs of neurological dysfunction attributable to a focal collection of blood within the brain parenchyma or ventricular system that is not caused by trauma [2]. Stroke being one of the major cause of disability has a higher impact on the society.

Stroke is an extremely rare event during pregnancy with the incidence ranging from 10 to 34/100,000 deliveries [3]. A recently conducted cohort in England showed that compared to non-pregnant women, stroke rates are lower during the antepartum period, 9-fold higher in the early peripartum period and 3-fold higher in the early postpartum period [4]. While the epidemiology of stroke in pregnancy in Africa and Cameroon remained largely unknown, the case fatality of stroke in Douala, Cameroon was estimated at 26.8% in a recent study by Mapoure et al [5].

The authors reports a case of a 32 year old female Cameroonian gravida 4 para 3 who presented at 34 weeks of gestation with sudden onset of right sided hemiplegia associated with headache, blurred vision and stage 2 hypertension. Cerebral (computed tomography) CT scan confirmed a left parietal spontaneous haemorrhage. Emergency caesarean delivery was done and the recovery uneventful. The aim of the case is to highlight the importance of rapid history and examination including neurological examination in pregnant women presenting with raised blood pressure. The authors adhered to the CARE guidelines/ methodology for reporting case reports [6].

### Case presentation

A 32 year old female Cameroonian gravida 4 para 3 at 34 weeks of gestation presented to the labour and delivery unit of Mbalmayo district hospital with 8 h history of severe generalized headache, expressive aphasia and right sided paralysis in an afebrile context. This was associated with blurred vision but no convulsions. There was no epigastric pain and no difficulty breathing and no history of trauma or fall. For this current pregnancy, antenatal care (ANC) was started at 18 weeks with a booking blood pressure of 100/70 mmHg. She did four ANCs and all were uneventful. During her routine four ANCs here blood pressure was always less than 140/90 mmHg and her urine dipsticks done during the four ANCs were all negative for proteinuria. She refused neurological symptoms such as headache during pregnancy. She has a history of gestational hypertension in her third pregnancy. There was no family history of chronic hypertension, diabetes and chronic kidney diseases. On examination she was afebrile with a blood pressure of 182/126 mmHg and pulse of 112beats/minute. Neurological examination revealed Glasgow coma score of 13/15, right sided hemiparesis and expressive Broca’s aphasia, no signs of meningeal irritation. The abdomen was distended by a gravid uterus with a fundal height of 35 cm, foetus in a longitudinal lie and cephalic presentation. The cervix was long, posterior, soft and closed with a station of − 1. We had a working diagnosis of severe pre-eclampsia complicated by stroke. Shown on Table 1 are laboratory investigations done and their results.

**Table 1:** Results of initial laboratory investigations

Investigation	Result	Reference value
Plasma glucose	5.3 mmol/L	3.9–6.1 mmol/L
Urea	1.2 mmol/L	2.0–7.1 mmol/L
Creatinine	70 μmol/L	50–110 μmol/L
Sodium	132 mEq/L	135-145 mEq/L
Potassium	3.8 mEq/L	3.5–5.5 mEq/L
Chloride	109 mEq/l	98-112 mEq/L
Alanine amino transferase (ALAT)	17 IU/L	10-40 IU/L
Aspartate amino transferase (ASAT)	16 IU/L	10-35 IU/L
Alkaline phosphatase	57 IU/L	36-92 IU/L
Urine dipstick for protein	3+	Negative
Haemoglobin level	11.8 g/dl	12 -16 g/dl
White cell count	9.800 cells / mm^3^	4.000–10.000 cells / mm^3^
Platelet count	160.000cells/mm^3^	150.000–400.000 cells / mm^3^

**Fig. 1:**
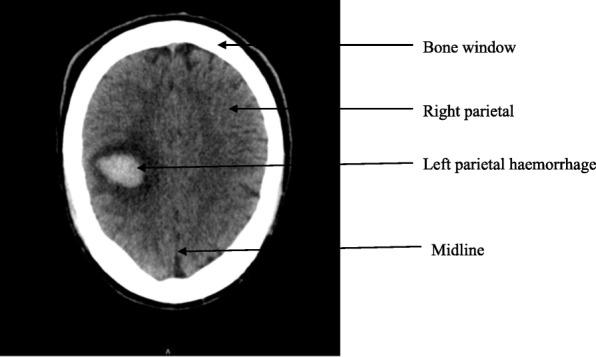
Emergency cerebral CT scan showing spontaneous left parietal haemorrhage

An emergency obstetric ultrasound showed a life foetus with an estimated foetal weight of 2300 g at 33 weeks of gestation. Emergency cerebral non contrast-CT scan showed a 3.2 cm hyperdense region in the left parietal lobe with surrounding hypodensity due to clot retraction as shown on Fig. 1. Emergency management by the obstetrician consisted of MgSO4 using the Pritchard protocol [7], which consisted of 14 g loading dose then 5 g maintenance every 6 h until 24 h after caeserean section; bethamethasone 12 mg intramuscular and reduction of blood pressure with nicardipine 5 mg/h. Four hours later an emergency caesarean section was done by the obstetrician under spinal anaesthesia and it let to the extraction of a life female with APGAR 8 and 10 at the 1st and 5th minute respectively and weight 2200 g. The management after caesarean section consisted of hospitalization in the intensive care unit with nicardipine titrated in an electric syringe at 2.5 mg/hour, ceftriaxone 2 g intravenous, Paractamol 1 g 8 hourly, and ringers lactate 6 hourly for 24 h. Post-operative management was done by a multidisciplinary team including a neurologist, cardiologist, intensive care physician, obstetrician, neonatologist and physiotherapist. On postoperative day 2 she was transferred from the intensive care unit to the maternity where she spends five additional days on nicardipine slow release 50 mg 12 hourly and paracetamol 1 g 8hourly and was later release after the ten days on nicardipine 50 mg daily and daily physiotherapy. Six weeks during routine postpartum visit the blood pressure was normal and patient was no longer aphasic and shet has regained the muscle strength partially. The baby was hospitalised in the neonatal unit for 10 days and discharged alongside the mother.

## Discussion and conclusions

Recent studies have demonstrated that stroke is a rare event during pregnancy with incidence relatively lower in the antepartum period compared to non-pregnant women [5]. In one study risk factors of stroke during pregnancy were found to include: sociodemographic factors such as age ≥ 35 years(*R* = 2.2, 95% CI = 1.2–2.2), African-American(OR = 1.5, 95% CI = 1.2–1.9); medical and obstetric determinants such as migraine headache (OR = 16.9, 95% CI =9.7–29.5), thrombophilia (OR = 16.0, 95% CI = 9.4–27.2), systemic lupus erythematosus (OR = 15.2, 95% = CI 7.4–31.2), heart disease (OR 13.2, 95% = CI 10.2–17.0), sickle cell disease (OR = 9.1, 95% CI = 3.7–22.2), hypertension (OR = 6.1, 95% CI 4.5–8.1) and thrombocytopenia (OR = 6.0, 95% = CI 1.5–24.1), postpartum haemorrhage (OR 1.8 95% CI = 1.2–2.8) preeclampsia and gestational hypertension (OR = 4.4 95% CI 3.6–5.4) [8]. Hypertension remain one of the main easily reversible risk factor and focus of treatment of stroke in pregnancy [9, 10]. In the indexed case presented, the only risk factor identified was pre-eclampsia / hypertension. Her haemoglobin electrophoresis was AA and platelet count normal, however due to the limited capacity of the laboratory we could not assess for thrombophilia and systemic lupus erythematosus.

Compared with women without hypertension, women with a hypertensive disorder in pregnancy are six- to nine-fold more likely to develop stroke in pregnancy [8, 11].The role of pre-eclampsia in stroke has earlier been described in a review in 2013 which showed that pre-eclampsia / eclampsia is usually associated with about a third of stroke cases in pregnancy [12]. The most common type of stroke associated with pre-eclampsia / eclampsia is haemorrhagic stroke [13, 14].

The diagnosis is usually confirmed by a cerebral non-contrast computed tomography (CT) scan which will show a region of spontaneous hyper-density in the case of haemorrhagic stroke [15]. Contrary to public opinions, cerebral CT scan is not contraindicated in pregnancy because the radiation doses exposed to during a cerebral CT scan are well below the threshold of risk of foetal teratogenicity [16]. Cerebral (magnetic resonance imaging) MRI is the imaging modality of choice in case of suspicion of acute ischaemic stroke [16–18]. The regular unavailability and high cost of this imaging modality limited it use in favour for a cerebral CT scan which is readily available and affordable in our resource low-setting. In the indexed case cerebral CT scan showed spontaneous intracerebral haemorrhage in the parietal lobe. The management of stroke in pregnancy is multidisciplinary involving the obstetrician, neurologist, neurosurgeon, intensivist, anaesthetist, physiotherapist and paediatrician.

The main aim of management is to maintain cerebral perfusion pressure prevent secondary brain injuries [17] and deliver the baby and the placenta. Blood pressure should be reduced judiciously in the acute phase with a target of ≤160/110 [19]. Intravenous labetalol has been widely used as first line for the reduction of blood pressure in patients with stroke during pregnancy [12]. MgSO4 should be added in the acute management for eclampsia prophylaxis [17]. Our indexed case described herein received intravenous nicardipine for control of blood pressure due to the unavailability of intravenous labetalol in our resource-limited setting and MgSO4 using the Pritchard protocol for prevention of seizures [7]. The timing and mode of delivery is influenced by fetal condition, gestational age and severity of associated preeclampsia [17]. Historically, caesarean delivery has been advocated and is increasingly being used to circumvent the potential risks during labour and delivery [20, 21]. This was the mode of delivery in our patient which let to the extraction of a life female baby. The prognosis of haemorrhagic stroke occurring during pregnancy is more severe compared to ischaemic stroke [22].

## Conclusion

Albeit the relative rarity of this condition in pregnancy, this case highlights the importance of a good neurological examination in a pregnant woman presenting with neurological symptoms and raised blood pressure. It also illustrate the importance of a well equip and multidisciplinary team in the management of emergencies. Finally it demonstrates the importance of early and aggressive management in life threatening conditions such as haemorrhagic stroke. The initiation of clinical registries are a potential avenue to increase awareness around these fatal conditions and thereby contribute to reduction of cardiovascular related morbidity and mortality. The author suggests a case control study should be carried out in order to well describe determinants of intracerebral haemorrhage in pregnancy.

## Abbreviations

ANC: Antenatal Care
CT Scan: Computed tomography scan
MRI: Magnetic resonance imaging
